# Interplay of *Plasmodium falciparum* and thrombin in brain endothelial barrier disruption

**DOI:** 10.1038/s41598-019-49530-1

**Published:** 2019-09-11

**Authors:** Marion Avril, Max Benjamin, Mary-Margaret Dols, Joseph D. Smith

**Affiliations:** 1 Seattle Children’s Research Institute, Seattle, WA 98109 USA; 2 Department of Global Health, University of Washington, Seattle, WA 98195 USA

**Keywords:** Mechanisms of disease, Parasite host response

## Abstract

Recent concepts suggest that both *Plasmodium falciparum* factors and coagulation contribute to endothelial activation and dysfunction in pediatric cerebral malaria (CM) pathology. However, there is still limited understanding of how these complex inflammatory stimuli are integrated by brain endothelial cells. In this study, we examined how mature-stage *P*. *falciparum* infected erythrocytes (IE) interact with tumor necrosis factor α (TNFα) and thrombin in the activation and permeability of primary human brain microvascular endothelial cell (HBMEC) monolayers. Whereas trophozoite-stage *P*. *falciparum*-IE have limited effect on the viability of HBMEC or the secretion of pro-inflammatory cytokines or chemokines, except at super physiological parasite-host cell ratios, schizont-stage *P*. *falciparum*-IE induced low levels of cell death. Additionally, schizont-stage parasites were more barrier disruptive than trophozoite-stage *P*. *falciparum*-IE and prolonged thrombin-induced barrier disruption in both resting and TNFα-activated HBMEC monolayers. These results provide evidence that parasite products and thrombin may interact to increase brain endothelial permeability.

## Introduction

Cerebral malaria (CM) is a life-threatening complication associated with the sequestration of *Plasmodium falciparum*-infected erythrocytes (IE) in the brain microcirculation^1–4^. In African children, CM is associated with endothelial activation and dysfunction^5^. Recent neuroimaging studies have implicated breakdown of blood brain barrier^6^ and severe brain swelling^7^ in disease etiology. The vascular dysfunction in cerebral malaria is believed to result from a combination of microvascular obstruction and tissue perfusion abnormalities^8,9^, altered coagulation^10,11^, systemic and local inflammatory processes^12^, and damaging parasite products (reviewed in Miller *et al*.^13^). From *in vitro* studies, numerous parasite products can activate endothelial cells or increase permeability, including *P*. *falciparum* histones^14^, malaria hemozoin pigment with parasite DNA^15^, and histidine-rich protein 2 (PfHRP2)^16^.

Recent findings suggest that coagulation may play an important role in pediatric CM pathogenesis. Pediatric CM autopsies have revealed fibrin deposition^5,11^, endothelial tissue factor expression^17^, and platelet recruitment^18,19^ in cerebral microvessels. Moreover, clinical laboratory evidence of disseminated intravascular coagulation is linked to a higher fatality risk^10^. Thrombin is a key enzyme in the coagulation cascade and a barrier permeability mediator^20^. Thrombin converts fibrinogen into fibrin and increases vascular permeability by cleaving protease activated receptor 1 (PAR-1) on endothelial cells^20^ leading to actin stress fiber formation and disassembly of cell junction proteins that open temporary gaps between cells^21^. Thrombin’s pro-inflammatory activities are countered by the activated protein C (APC) pathway^22^. Protein C is a serum protein that binds to endothelial protein C receptor (EPCR) on the endothelial surface and becomes activated by the thrombin-thrombomodulin complex^23^. APC counteracts thrombin signaling by inhibiting its activation and by upregulating anti-inflammatory, anti-apoptotic, and barrier restorative signals in endothelial cells^22^. Inflammation shifts the hemostatic mechanisms in favor of thrombosis, and impaired thrombin regulation is implicated in endothelial dysfunction from sepsis and other inflammatory diseases^24^.

Several findings suggest that the APC pathway is compromised in CM (reviewed in Bernabeu *et al*.^25^) due to a combination of loss of EPCR on microvascular endothelial cells^11^ and parasite interference of the APC-EPCR interaction^26^. Parasite sequestration is mediated by specific interactions between members of the clonally variant *var* gene/*P*. *falciparum* erythrocyte membrane protein 1 (PfEMP1) family and receptors on the host vascular endothelium^27–29^. EPCR-binding parasites have affinity for human brain endothelial cells *in vitro*^30,31^ and are increased in cerebral malaria patients^26,32–34^. Moreover, EPCR-binding *var* transcripts are linked to severe brain swelling in CM^35^ and recombinant parasite domains compete with APC for binding to EPCR^26,36–39^. Collectively, these findings strongly implicate both parasite factors and pro-coagulant processes in cerebral malaria pathology, although the molecular mechanisms of how these complex inflammatory stimuli translate into endothelial activation and blood-brain barrier dysfunction remain only partially understood.

In this study, we investigated the interaction of mature *P*. *falciparum*-IE (trophozoite and schizont stages) and thrombin in co-culture models with primary human brain microvascular endothelial cells (HBMEC). Our study suggests that mature-stage parasites differ in their barrier-disruptive activity and that schizont-stage *P*. *falciparum*-IE delay endothelial cell recovery from thrombin-induced barrier disruption.

## Results

### Comparative evaluation of immortalized and primary brain endothelial cells for thrombin-induced barrier permeability assays

To investigate the interaction of *P*. *falciparum*-IE and thrombin in brain endothelial activation and permeability, we first compared surface expression levels of parasite cytoadhesion receptors and the main thrombin receptor PAR-1 on an immortalized and primary brain endothelial cells. Compared to an immortalized brain endothelial cell line (THBMEC^40^), resting HBMEC expressed higher levels of EPCR (MFI 6041 versus 761), higher levels of PAR-1 (MFI 365 versus 126), and lower levels of ICAM-1 (MFI 29 versus 391) (Supplementary Fig. S1A). In addition, HBMEC were 100% reactive for the endothelial marker, CD31, and upregulated ICAM-1 following TNFα stimulation (MFI 2206) (Supplementary Fig. S1A).

We then compared THBMEC and primary HBMEC for thrombin-induced barrier disruption by xCELLigence Real-Time Cell Analysis (Supplementary Fig. S1B). Following seeding at a similar cell density, both cell types reached a confluency plateau by approximately 18–20 hrs. However, the raw cell index (CI), a quantitative measure of the number of cells, was lower for THBMEC than HBMEC (Supplementary Fig. S1B), likely due to THBMEC having a longer and thinner cell shape. In the xCELLigence assay, relative changes in cell morphology or cell adhesion following treatment with barrier permeability mediators are detected as a reduction of CI. As expected, primary HBMEC responded to 10 nM thrombin treatment with the characteristic rapid decrease in CI that reached a maximum by 15–20 min and returned to baseline by 1 hr. By comparison, thrombin induced a small and extremely truncated (~1 min) decline in CI in THBMEC (Supplementary Fig. S1B, lower right corner), likely due to the very low PAR-1 surface expression in the immortalized brain cells, as low and high doses of thrombin elicit opposing barrier strengthening or barrier disruptive responses in endothelial cells^41^. Consequently, primary HBMEC were used for the remainder of experiments.

Next, to be able to study whether parasite factors modify thrombin-induced permeability, we identified a concentration of thrombin that would induce a partial barrier disruption. We compared serum-free or 5% serum medium assay conditions because serum contains anti-thrombin factors. We observed a dose-dependent change in CI when comparing 2.5, 5 or 10 nM of thrombin in both conditions (Supplementary Fig. S1C) and choose 5 nM for the remainder of experiments.

### TNFα modifies expression levels of EPCR, ICAM-1, and PAR-1 in primary HBMEC

**Figure 1:**
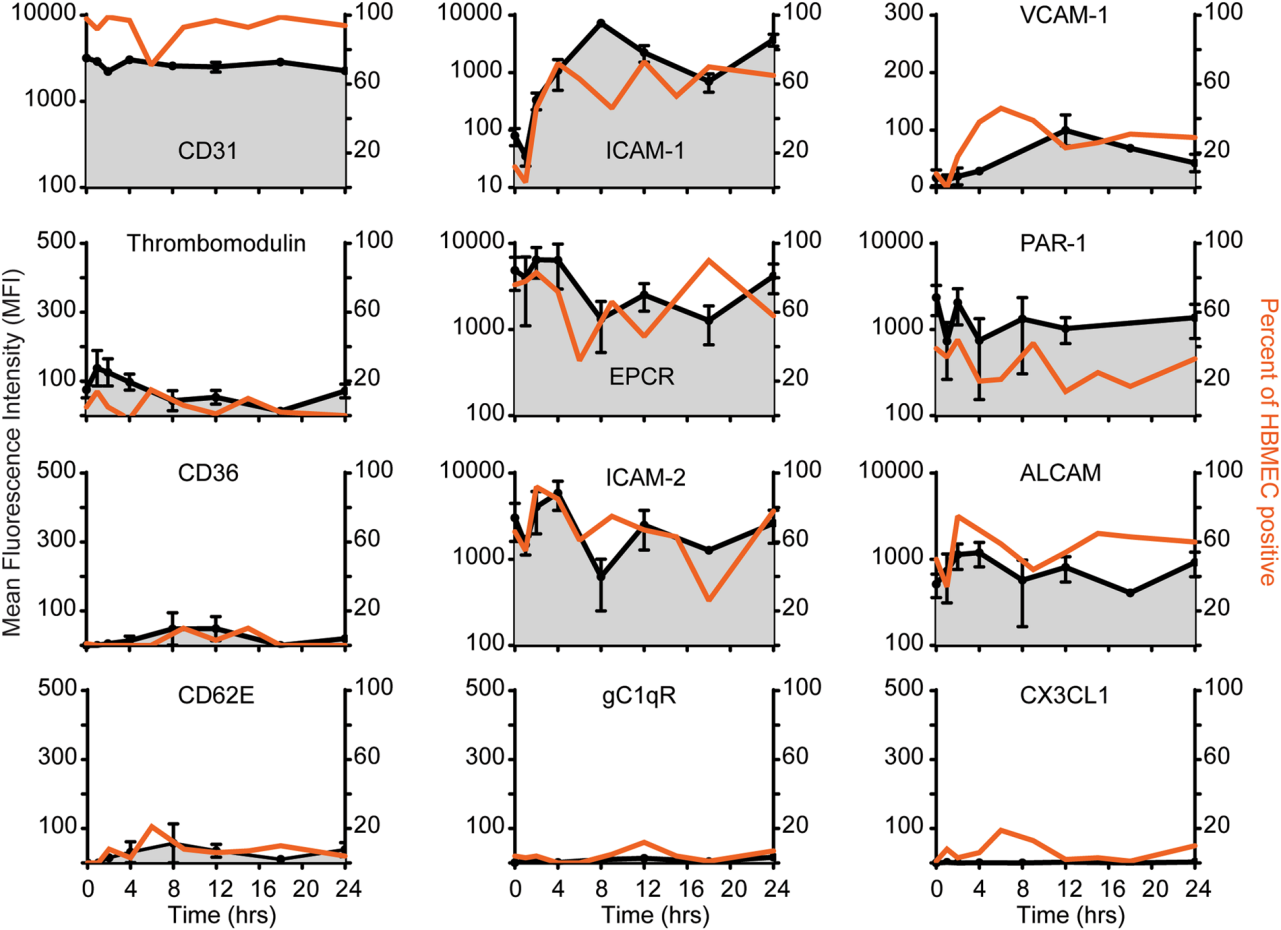
Endothelial surface receptor expression following TNFα time course stimulation. The black line shows the corrected geometric mean fluorescence intensity (MFI specific antibody minus MFI appropriate antibody control). The orange line shows the percent of HBMEC gated reactive for each surface receptor. Time 0 corresponds to unstimulated HBMEC.

Because pro-inflammatory cytokines can modify the surface levels of cell adhesion molecules and receptors regulating coagulation^42,43^, we performed a TNFα time course stimulation assay with primary HBMEC (Fig. 1 and Supplementary Fig. S2). The endothelial marker CD31 was expressed by nearly all resting cells and did not change significantly with TNFα stimulation (Fig. 1). As expected, ICAM-1 and VCAM-1 expression levels were highly upregulated by TNFα with slightly delayed kinetics for VCAM-1. By 24 hrs after TNFα stimulation, ICAM-1 surface levels were approximately 50 times higher and the percentage of positive cells had increased from 10% to 60%. Conversely, other known parasite binding partners, including CD36^44^, gC1qR1^45^, E-selectin (CD62E)^46^, and fractalkine (CX3CL1)^47^ were expressed at negligible levels on HBMEC cells, and ICAM-2^48^ levels fluctuated following TNFα treatment (Fig. 1). Of the receptors involved in thrombin signaling, thrombomodulin was expressed by few HBMEC, consistent with previous reports^37^, and EPCR and PAR-1 surface levels initially decreased and then rebounded (Fig. 1). Since the expression levels were dynamic following TNFα activation, we chose to pre-stimulate HBMEC for 24 hrs, at which point ICAM-1 was highly upregulated and PAR-1 and EPCR expression were nearly equivalent to resting baseline levels.

### *P*. *falciparum*-IE induce minimal apoptosis of HBMEC

**Figure 2:**
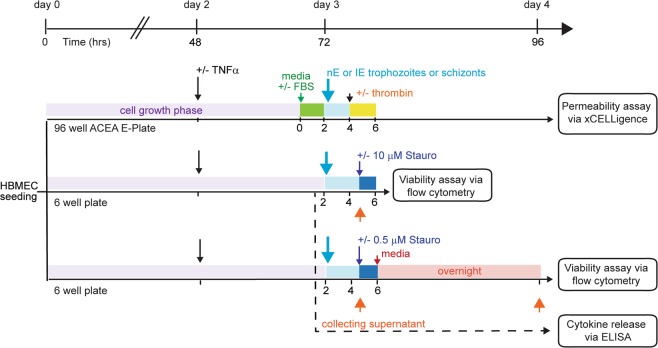
Overview of experimental design. For each experiment, parallel assays were performed on the same HBMEC culture to measure permeability, viability, and cytokine release. On day 0, from a single culture of primary HBMEC, cells were seeded into a 96 well ACEA E-plate for the permeability assay and into 6 well plates for the viability/apoptosis assays. Cells were grown until confluent on day 3. On day 2, 10 ng/ml of TNFα was added to stimulate the cells. The time frames in which media was replaced (+ or − FBS) or parasites and thrombin were added are indicated by the different colored areas. Time point collection of the supernatant for the ELISA assays are indicated by orange arrowheads.

We designed our experiments to simultaneously measure endothelium viability, cellular cytokine response, and permeability from the same HBMEC cultures under different environmental conditions (Fig. 2). To investigate the impact of *P*. *falciparum*-IE on HBMEC viability, we compared different parasite binding variants at two development stages. In particular, we compared an EPCR-binding parasite line (IT4var19), a dual EPCR and ICAM-1 binder (HB3var03), and a dual CD36 and ICAM-1 binding parasite line (ItG-ICAM-1)^30,49–51^ at the trophozoite-stage for either a short term (4–6 hrs) or long-term culture (12–20 hrs) or at the schizont-stage IE for a short term (4–6 hrs) (Fig. 2).

**Figure 3:**
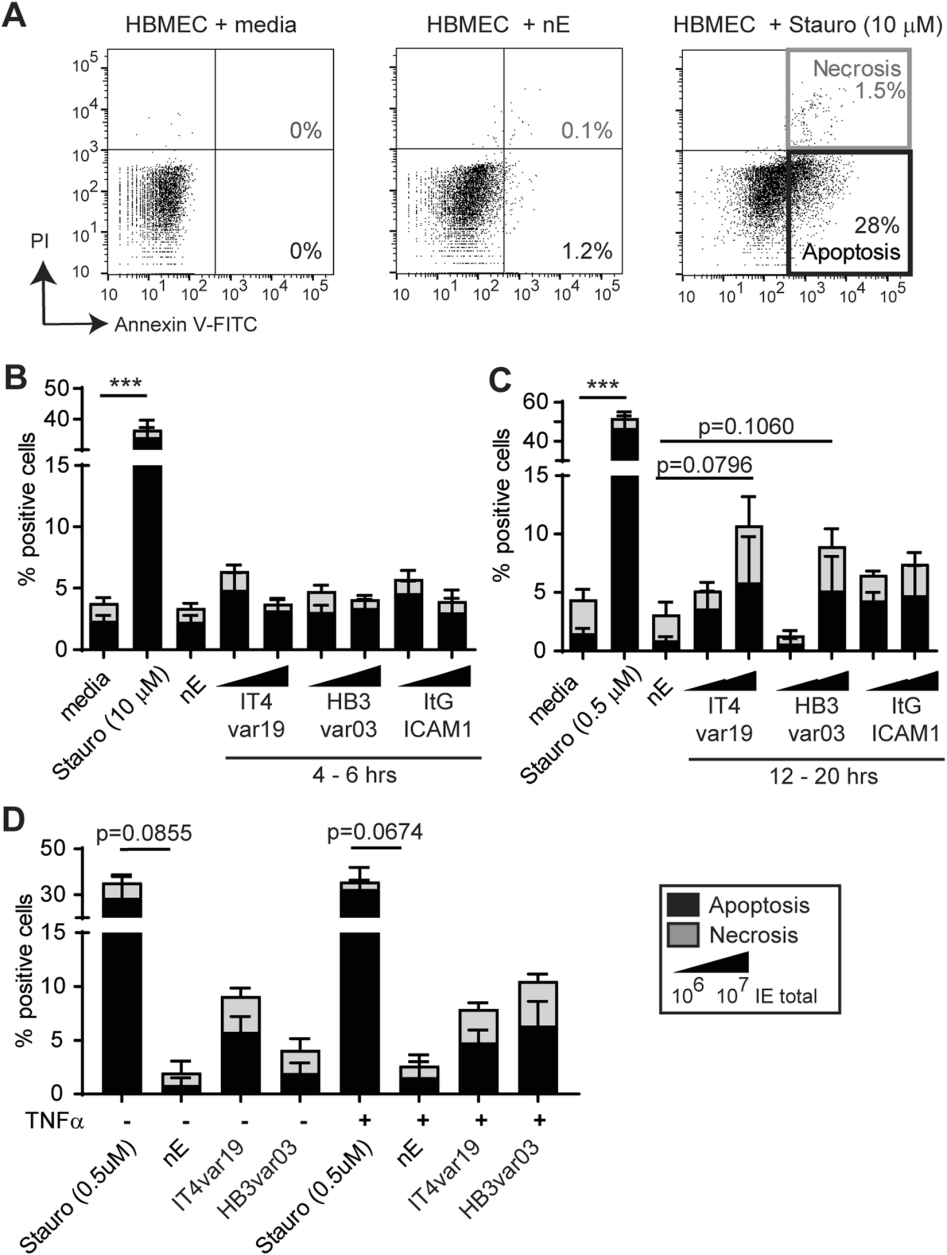
The induction of apoptosis in HBMEC co-cultured with *P*. *falciparum* IE. (**A**) Representative flow cytometry analysis of HBMEC stained with Annexin V-FITC and PI, with gating strategies and dot plots shown. (**B**–**D**) HBMEC were co-incubated with two concentrations of trophozoite-stage IE for 4–6 hrs (B) or overnight for 12–20 hrs (**C**) or with enriched schizont stage IE for 4–12 hrs (**D**). The percentage of apoptotic cells was determined by calculating Annexin V-positive cells (apoptotic, black) and PI-positive cells (necrotic, grey) from 10,000 cells analyzed. The results are expressed as mean values and standard error means (SEM). Data are from N ≥ 4 independent experiments with trophozoite-stage IE and N = 3 independent experiments for schizont-stage IE. Data were analyzed by Kruskal-Wallis one-way analysis of variance corrected by method of Benjamini, Krieger and Yekutieli. ***p = 0.001.

HBMEC viability was assessed by annexin-V and propidium iodide (PI) staining at two different cell ratios of trophozoite-stage IE (8–10:1 or 80–120:1 IE:HBMEC ratio) (Fig. 3A). For positive control, the kinase inhibitor staurosporine caused a significant percentage of apoptotic cells (34 ± 5% after 1 hr at 10 μM or 47 ± 8% after 12 hrs at 0.5 μM) (Fig. 3A–C). In short term co-cultures, trophozoite-stage parasite lines induced minimal cell death (3–5%), compared to nE controls (2 ± 0.5%) (Fig. 3B). In overnight cultures, there was a parasite dose-dependent increase in endothelial cell death (9–10%) (Fig. 3C). Similarly, schizont-stage IE (20:1 IE:HBMEC ratio) induced a low level of cell death (5–10%) of both resting or TNFα-activated HBMEC, although the difference did not reach statistical significance (Fig. 3D). Taken together, this analysis suggests that late-stage IE may cause low levels of cell death in both resting and TNFα stimulated HBMEC.

### *P*. *falciparum*-IE do not induce major pro-inflammatory cytokine release from HBMEC

**Figure 4:**
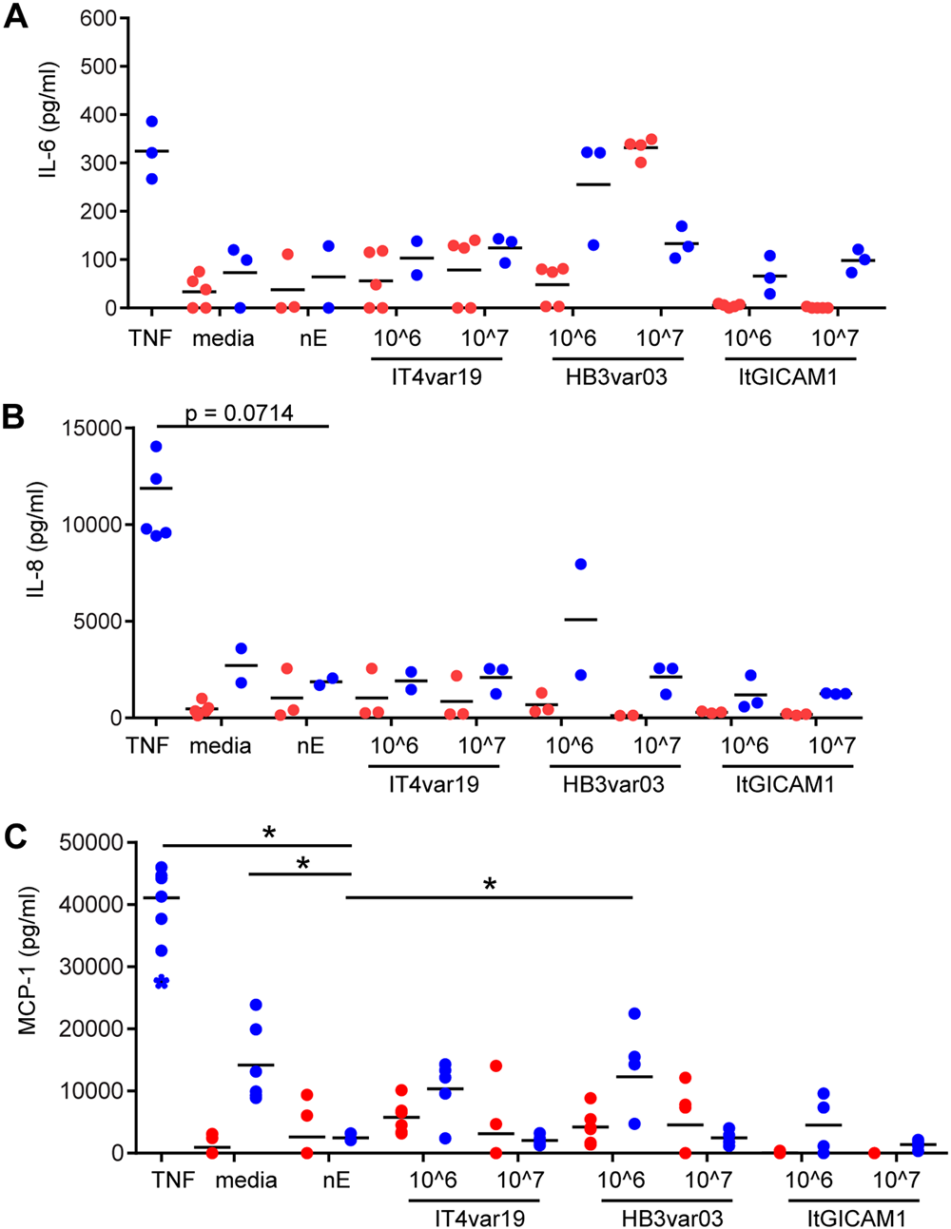
The induction of pro-inflammatory cytokines in co-cultures of HBMEC and *P*. *falciparum* IE. Concentration (pg/ml) of IL-6 (**A**) IL-8 (**B**) and MCP-1 (**C**) upon stimulation for 24 hrs with TNFα or co-cultured with nE or trophozoite-stage IE. Culture supernatants from short-term (4–6 hrs) incubations are shown in red and from overnight (12–20 hrs) incubations in blue. Symbols represent each experimental replicate, bars represent the mean. Data are from N = 3 independent experiments (**A**,**B**) or N ≥ 3 independent experiments (**C**) and were analyzed by Mann-Whitney non-parametric unpaired, two-tailed t test, relative to nE. *p < 0.05.

Previous work suggests that *P*. *falciparum* can stimulate dermal and lung endothelial cells to release IL-6, IL-8, and MCP-1^52–54^. To investigate whether *P*. *falciparum*-IE can trigger pro-inflammatory cytokine or chemokine production from primary HBMEC, supernatants were collected from the trophozoite-stage IE-HBMEC co-cultures at the end of the short-term (4–6 hrs) and long-term (12–20 hrs) incubations. TNFα stimulated production of IL-6, IL-8, and MCP-1 from HBMEC at 24 hrs was used as positive control (Fig. 4). Compared to nE, neither IT4var19 nor ItG-ICAM-1 trophozoite-stage IE induced secretion of IL-6 or IL-8 from HBMEC. However, HB3var03-HBMEC co-cultures had a small but significant secretion of IL-6 comparable to TNFα stimulation (Fig. 4A). Additionally, the chemokine MCP-1 was constitutively released by HBMEC and its secretion was similar or reduced in the presence of either nE or *P*. *falciparum*-IE (Fig. 4C).

### Interaction of trophozoite-stage IE and thrombin on HBMEC barrier properties

**Figure 5:**
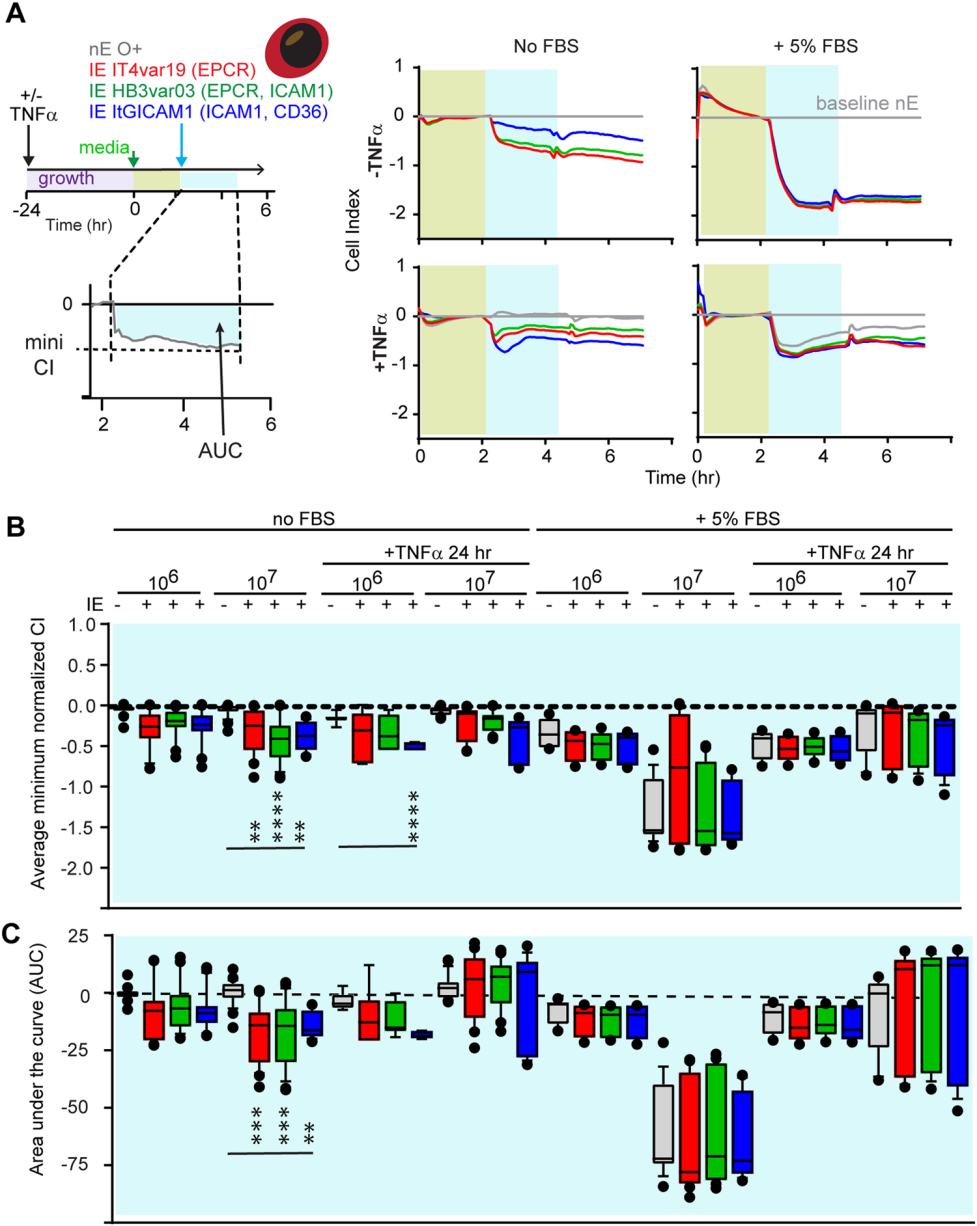
The induction of barrier disruption in HBMEC co-cultured with trophozoite-stage IE depends on culture conditions. (**A**) Schematic of the experimental design and a representative recording data in the xCELLigence assay after the addition of nE (grey) or IE (IT4var19, red; HB3var03, green; ItG-ICAM-1, blue) to the HBMEC monolayer. The green time frame indicates the 2 hrs window after HBMEC were switched to serum free media or maintained in 5% FBS. The first 2 hrs after parasite addition (blue timeframe) and subsequent 2 hrs period (white timeframe) are indicated. The traces on the left and right side show a side by side comparison of the normalized cell index (CI) in serum-free media (no FBS) or serum complemented media (+5% FBS) on resting (-TNFα) or stimulated (+TNFα) HBMEC. The minimum normalized CI for each condition is shown in (**B**) and the area under the curve (AUC) for the first 2 hrs of co-culture (blue timeframe) is shown in (**C**). The results are expressed as mean values (horizontal line) and interquartile ranges. Data are from N = triplicate wells of 4–10 independent experiments. **p = 0.01, ***p = 0.001, ****p = 0.0001.

Leukocyte engagement and cross-linking of ICAM-1 on endothelial cells enhances permeability^55–58^. To study whether *P*. *falciparum*-IE may also increase barrier permeability by this mechanism, we compared parasite lines with low and high ICAM-1 binding activity (HB3var03 and ItG-ICAM-1, respectively)^51^ to a non-ICAM-1 binder (IT4var19). Using the xCELLigence assay (Supplementary Fig. S3) we found that in resting HBMEC switched to serum-free assay conditions 2 hrs prior to parasite addition (Fig. 5A schematic), all three trophozoite-stage parasite lines induced a statistically significant and dose-dependent reduction in CI values, compared to control nE (Fig. 5B,C). Of the three parasite lines, the high ICAM-1 binding activity ItG-ICAM-1 parasite line was the most barrier disruptive on TNFα stimulated HBMEC (Fig. 5A–C). The same trend was apparent at both the low and high parasite densities, although the difference was statistically significant only at the lower IE-HBMEC ratio (Fig. 5B,C). Of interest, the endothelial barrier properties recovered more rapidly in TNFα-stimulated HBMEC than resting cells for all three parasite lines (Fig. 5A).

We also observed an influence of serum conditions with higher parasite-induced barrier disruption of resting HBMEC that were maintained in serum-supplemented media (+5% FBS) compared to serum-free medium (compare left and right recordings Fig. 5A). However, in the presence of serum, there was no difference between nE and *P*. *falciparum*-IE in barrier disruption (Fig. 5). By comparison, the extent of barrier disruption in activated HBMEC was similar in the serum-free and 5% serum conditions (compare left and right recordings Fig. 5A).

**Figure 6:**
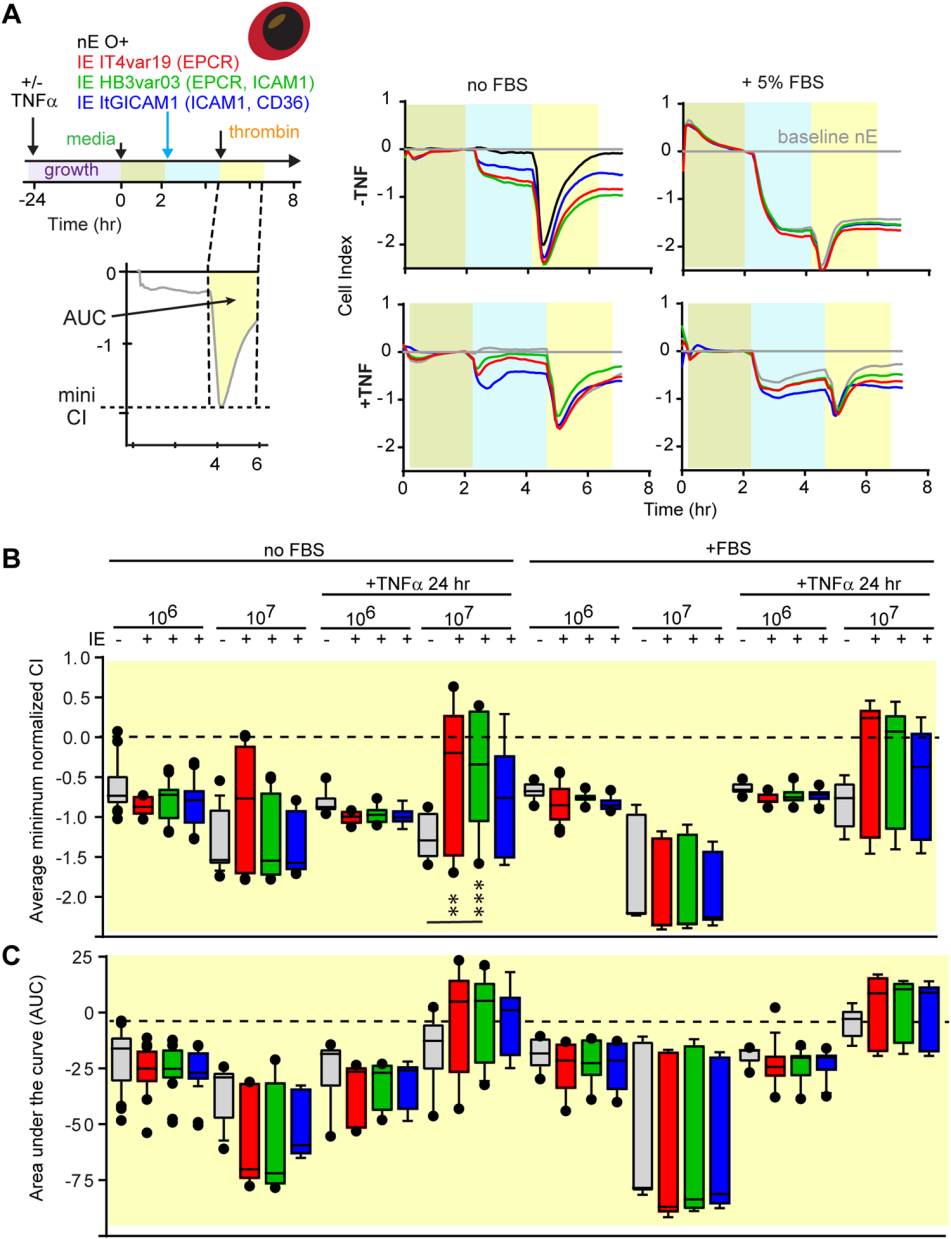
Barrier disturbance in HBMEC following combined stimulation with trophozoite-stage IE and thrombin treatment. (**A**) Schematic of the experimental design and a representative recording data in the xCELLigence assay following the addition of 5 nM thrombin in presence of nE (grey traces) or IE (IT4var19 red, HB3var03 green, ItG-ICAM-1 blue traces). The blue timeframe (first 2 hrs after parasite addition) is from the same experiment as Fig. 5. The yellow timeframe indicates the subsequent 2 hrs period after the addition of thrombin. The traces on the left and right side show a side by side comparison of the normalized CI in serum-free media (no FBS) or serum complemented media (+5% FBS) on resting (-TNFα) or stimulated (+TNFα) HBMEC. The minimum normalized CI for each condition is shown in (**B**) and the area under the curve (AUC) for the 2 hrs window following thrombin treatment is shown in (**C**). The results are expressed as mean values (horizontal line) and interquartile ranges. Data are from N = triplicate wells of 4–10 independent experiments. **p = 0.01, ***p = 0.001.

To study whether trophozoite-stage-IE modify thrombin-induced barrier permeability, 5 nM thrombin was added two hrs after *P*. *falciparum*-IE. Thrombin induced a characteristic rapid loss of barrier properties followed by a recovery in approximately 60–90 mins (Fig. 6A). In both resting and TNFα activated cells, thrombin was additive to the earlier disruption observed with parasites, so the total disruption was larger in monolayers receiving the higher parasite density (Fig. 6B,C). However, the magnitude of the thrombin-induced barrier disruption and the kinetics of barrier recovery were not altered by trophozoite-stage IE. Although thrombin was less disruptive on activated than resting HBMEC monolayers (Fig. 6), the percentage of PAR-1 positive HBMEC was lower at 24 hrs after TNFα stimulation (Fig. 1). As expected, thrombin was less barrier disruptive in the presence of 5% serum (Fig. 6B,C), likely due to anti-thrombin factors in serum. However, as with the serum-free assay conditions, trophozoite-stage IE did not increase the magnitude of thrombin-induced barrier disruption or delay the recovery.

### Interaction of schizont-stage IE and thrombin on HBMEC vascular permeability

**Figure 7:**
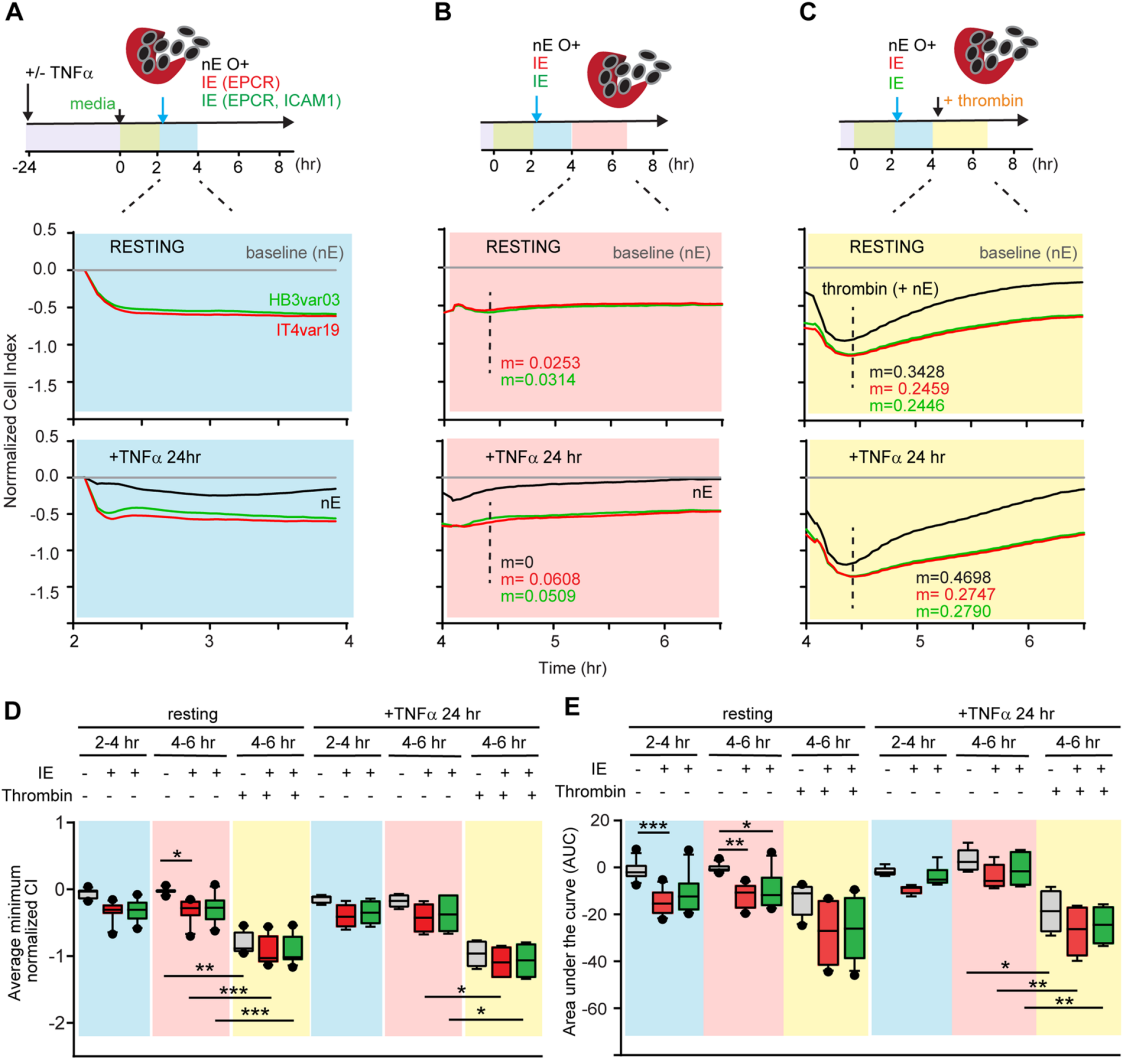
Barrier disturbance in HBMEC following combined stimulation with schizont-stage IE and thrombin treatment. (**A**–**C**) The top shows a schematic of the experimental design and bottom shows representative recording data in the initial 2 hrs period following schizont-stage *P*. *falciparum*-IE addition (blue window) (**A**) or in the subsequent 2 hrs period with either no thrombin addition (pink window) (B) or addition of 5 nM thrombin (yellow window). (**C**) The xCELLigence recordings are shown for nE (grey traces) or enriched schizont-stage IE (IT4var19 red traces, HB3var03 green traces). Experiments were conducted in serum-free media on resting (-TNFα) or stimulated (+TNFα) HBMEC. The minimum normalized CI for each condition is shown in (**D**) and the area under the curve (AUC) is shown in (**E**). The results are expressed as mean values (horizontal line) and interquartile ranges. Data are from N = triplicate wells of 4 independent experiments (-TNFα) or N = triplicate wells of 2 independent experiments (+TNFα). *p = 0.05, **p = 0.01, ***p = 0.001.

Previous work suggests that schizonts release bioactive products during red blood cell rupture that activate endothelial cells and increase permeability^14–16^. To investigate whether late stage parasite products interact with thrombin in endothelial permeability, we incubated HBMEC with enriched schizont-stage-IE (1–10:1 ratio, IE:HBMEC). On their own, *P*. *falciparum*-IE induced a rapid CI decrease in HBMEC that was sustained over the next 4 hrs (Fig. 7A,B). The magnitude of the parasite-induced CI decrease was similar on resting and TNFα activated cells (Fig. 7A,B). Notably, schizont-stage IE induced equivalent barrier disruption to trophozoite-stage IE at a 10-fold lower IE:HBMEC ratio (compare Figs 5 and 7).

To study whether schizont-stage parasites modify thrombin-induced permeability, 5 nM thrombin was added two hrs after *P*. *falciparum*-IE. The effect of thrombin was additive to the earlier disruption observed with schizont-stage IE (Fig. 7A–C). However, in contrast to trophozoite-stage IE, thrombin-induced barrier recovery was delayed, as indicated by a slower slope of recovery compared to nE (Fig. 7C). The delay in barrier recovery following thrombin treatment was observed in both resting and TNFα-activated HBMEC (Fig. 7C), indicating that schizont-stage IE prolong thrombin-induced barrier disruption.

## Discussion

During infection, endothelial cells integrate cues from a variety of pro-inflammatory and coagulation factors to effectively respond to pathogens. Overstimulation of endothelial cells in response to inflammation can lead to maladapted microvascular environments^24^. From pediatric CM autopsy studies, sequestered IE, thrombin and/or fibrin are sometimes co-localized in cerebral microvessels^5,59^. However, little is known about how parasite factors and host inflammatory products may interact to cause endothelial dysfunction in CM. The aim of our present study was to investigate the interaction of *P*. *falciparum*-IE and thrombin on resting and activated primary human brain endothelial cells.

*In vitro* models have suggested that *P*. *falciparum*-IE can activate endothelial cell monolayers to secrete pro-inflammatory cytokines^52–54,60^, induce apoptosis^60–64^, or increase vascular permeability^16,62,65^. However, much of this analysis has been conducted on immortalized brain endothelial cell models^16,61–64^ and less work has been done on primary brain microvascular endothelial cells^65,66^. Multiple mechanisms have been proposed by which *P*. *falciparum*-IE may activate endothelial cells including pathways at early time points following parasite adhesion, such as release of IE-derived extracellular vesicles containing functional miRNA-Argonaute 2 complexes^67^ or cross-linking of adhesion receptors^68^, or later events associated with rupture of IE^14,16^. Here, we compared trophozoite or schizont-stage IE in co-cultures with primary HBMEC. Whereas *P*. *falciparum*-IE can induce IL-6, IL-8, and MCP-1 in primary human dermal and lung microvascular endothelial cells^14,69^, we observed a very low and dose-dependent release of IL-6 in primary HBMEC to only one of the three parasite lines. The difference in findings could relate to endothelial cell type differences or parasite-dependencies, as cytokine and chemokine secretion was also a variable phenotype for different parasite isolates co-cultured with dermal endothelial cells^69^. Of interest, there was also a relatively low cell death in primary HBMEC compared to previous work on immortalized brain endothelial cells showing much higher cell death^61,62^ and increased death following TNFα activation^64^. There are several possible explanations for these discrepancies, including that the susceptibility to apoptosis has been found to differ between primary pulmonary endothelial cells and immortalized brain endothelial cells^61,63^. Moreover, the cell death inducing phenotype differs between parasite isolates^61,63,70,71^ and is influenced by the buffering capacity of culture media^63^. Thus, both assay conditions and cell and parasite models may influence the endothelial cell death phenotype.

Our main finding relates to the interaction between parasites and thrombin in HBMEC barrier disruption. Thrombin’s activity is under tight regulation with multiple feedback mechanisms to control thrombosis. Although previous work has revealed cross-talk between inflammatory cytokines and thrombosis^24^, there has been little investigation into whether parasites factors interact with thrombin to exacerbate endothelial dysfunction. Using primary HBMEC, we show that schizont-stage parasites cause higher barrier disruption than trophozoite-stage parasites, even at 10-fold lower parasite concentrations. Of interest, the extent of parasite-induced barrier disruption was influenced by serum assay conditions. Further work is needed to more fully understand how serum withdrawal may have interacted with parasites to induce vascular permeability in HBMEC. However, serum contains growth factors, and serum withdrawal can trigger stress responses in cells, including the induction of reactive oxygen species response within 3–4 hrs^72,73^. Unlike trophozoites, schizont-stage parasites interacted to prolong thrombin-induced barrier disruption, and thus may act like pro-inflammatory cytokines that have previously been shown to extend thrombin-induced barrier disruption in endothelial cells^74^. Although the underlying mechanism(s) of the interaction remain to be determined, these findings have important implications for disease mechanisms in cerebral malaria pathogenesis, since both thrombin and sequestered IE are frequently co-localized in pediatric CM brain autopsy specimens^59^. Thus, this finding raises the possibility that barrier-perturbing factors released by late stage *P*. *falciparum*-IE may interact with thrombin to amplify endothelial dysfunction in cerebral malaria. A limitation of this work is that it was performed on endothelial monolayers, which differ from intact microvessels^75^. Nevertheless, significant molecular insights into barrier regulation have been gained from endothelial monolayer models^21^.

**Figure 8:**
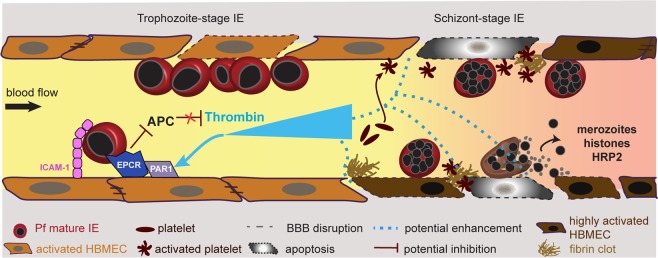
Proposed model for disruption of BBB caused by interaction between parasite and host factors. Sequestration of trophozoite-stage IE may induce minimal brain endothelial cell death or permeability on their own, unless at super physiological concentration, and did not amplify thrombin-induced barrier disruption. By comparison, sequestered schizont stage IE may induce low levels of cell death and permeability, and also delayed barrier recovery from thrombin, which could exacerbate localized endothelial dysfunction in CM. Additionally, parasite cytoadhesion to EPCR on endothelial cells may impair the cytoprotective and barrier enhancing APC pathway, which normally counteracts thrombin-induced barrier disruption.

Taken together, our findings support a model (Fig. 8) in which sequestration of early stage trophozoite IE causes minimal endothelial cell death or permeability on their own, especially at lower parasitemia. By comparison, sequestered late-stage IE may increase localized endothelial dysfunction, by releasing barrier disruptive factors that interact with thrombin in increasing barrier permeability. Moreover, when sequestered IE engage EPCR on brain endothelial cells this may further imbalance microvascular hemostasis by impairing the cytoprotective and barrier restorative APC-EPCR interaction that normally counteracts thrombin and other inflammatory stimuli^26,37–39^. In summary, these findings suggest potential interactions between thrombin and sequestered parasites in endothelial dysfunction.

## Methods

### Human microvascular endothelial cell cultures

Transformed brain endothelial cells (THBMEC), provided by Dr. Monique Stins (Johns Hopkins Bloomberg School of Public Health), were used from passage 38 to 39 and cultured as previously reported^30,40,76^. Primary human brain microvascular endothelial cells (HBMEC, ACBRI376) were purchased from Cell Systems at passage 3. Cells were expanded in Endothelial basal medium (EBM) in accordance with manufacturer specifications on an extracellular matrix-coated surface (Attachment Factor, Cell Systems) and frozen at passage 5. Cells were used in experiments from passage 5 to 12 (average 8–9) in Endothelial Cell Growth Medium MV2 (EGM-2 MV Bulletkit) with 5% FBS and supplements provided by PromoCell. HBMEC were certified positive by the manufacturer for expression of Von Willebrand factor, for acetylated low-density lipoprotein uptake, and for CD31 expression. HBMEC were routinely monitored for CD31 by flow cytometry. All cells tested negative for mycoplasma.

### Surface receptor labelling of human brain endothelial cells

THBMEC or HBMEC cells were seeded on collagen coated flasks and cultured for three to four days until confluent. Cells were rinsed with HBSS and then lifted with 15 mM EDTA in HBSS. Then 5 × 10^4^ cells were distributed into wells of a 96 well plate and washed 1X with PBS (0.5% BSA) at room temperature. Antibody labelling was performed on ice for 30 min each step, using the following: goat anti-human CX3CL1/Fractalkine (15 μg/ml, R&D Systems, AF365) or goat anti-human EPCR (30 μg/ml, R&D Systems, AF2245) followed by rabbit anti-goat Alexa488 coupled antibodies (A-21467, Molecular Probes, 1/400). Mouse anti-human VCAM-1/CD106 (10 μl for 10^5^ cells, R&D Systems, FAB5649P) followed by goat anti-mouse Alexa488 coupled antibodies (Molecular Probes, 1/200). Mouse anti-human CD31 (BD Pharmingen, mAb, 560983, clone WM59) or mouse anti-human ICAM-1 (0.5 μg/ml, Abcam, ab19756) were detected by PE-labelled mouse monoclonal IgG1 (2 μl for 10^5^ cells, Abcam, ab91357). Directly conjugated Alexa488 or FITC mouse monoclonal isotype control IgG1 (2 μg/ml, Abcam, ab106163) were used to control for labeling by mouse anti-human PAR-1 Alexa488-conjugated antibody (0.5 μl for 10^5^ cells, R&D systems, FAB3855G), mouse anti-human CD36 FITC-conjugated antibody (10 μl for 10^5^ cells, Abcam, ab39022), mouse anti-human ICAM-2 (2 μg/ml, Abcam, ab47207), mouse anti-human thrombomodulin FITC-conjugated antibody (1 μl for 10^5^ cells, Abcam, ab27396), and mouse anti-human gC1qR FITC-conjugated antibody (1/50, Abcam, ab125138). For assays with proinflammatory pre-stimulation, confluent cell monolayers were stimulated with 10 ng/ml TNFα (Sigma, T0157) for 24 hrs at 37 °C. For the TNFα time course experiment, HBMEC were analyzed at 1, 2, 4, 8, 12, 18 and 24 hrs. Results were expressed relative to control (no treatment) and the percentage of positive gated cells was reported. Prior to analysis in an LSRII (Becton Dickinson), cells were fixed with 2% w/v of paraformaldehyde for 10 min and 15,000 single live cells were gated using the Live/Dead fixable violet Dead cell stain kit (Molecular Probes, L34955). Data was analyzed using FLOWJO v10 software (Tree Star Inc.).

### Apoptosis assays

Mature trophozoite-stage or schizont-stage enriched IE cultures were incubated with HBMEC monolayers for 4–6 or 12–20 hrs intervals at ratios ranging from 8–12 (10^6^) to 80–120 (10^7^) IE per endothelial cell. As a control, 10 μM or 0.5 μM staurosporine (Abcam, ab120056) was added for 1 or 4 hrs to trigger HBMEC apoptosis. For assays with TNFα pre-stimulation, cells were stimulated with 10 ng/ml TNFα (Sigma, T0157) for 24 hrs at 37 °C. For flow analysis, cells were lifted with trypsin/EDTA solution which also led to the detachment of most of the bound IE. We then labeled with Annexin V and PI according to manufacturer’s specifications (Abcam, ab14085). As red blood cells are much smaller than endothelial cells, the following cytometer settings (FSC 130 and SSC 150) were applied to gate only for the endothelial cells. Relative Annexin V/PI fluorescence was measured by flow cytometry on a LSRII cell sorter (Becton Dickinson) and analyzed using FLOWJO V10 software (Tree Star Inc.).

### Production of cytokines by HBMEC

The supernatants from wells of IE:HBMEC co-cultures used above for the apoptosis assays were harvested before the wash steps and stored at −20 °C. Cytokine levels were quantified by sandwich ELISA kits for human IL-6 (ThermoFisher, 88–7066), IL-8 (CXCL8) (ThermoFisher, 88–8086) and MCP-1 (CCL2) (ThermoFisher, 88–7399) following the manufacturer’s instructions. The culture supernatants were diluted in a final volume of 0.1 ml as follows: 1/2 for IL-6, 1/40 for IL-8 and 1/40 for MCP-1, and their respective concentration in pg/ml was determined using standard curves. For positive control, the level of cytokine secretion by HBMEC was measured following pre-stimulation with 10 ng/ml TNFα for 24 hrs. For negative control, supernatants from co-incubation with either media alone or normal O^+^ erythrocytes (nE O+) were used.

### Parasite lines

*P*. *falciparum* parasites were cultured using human O+ erythrocytes in the presence of 10% pooled human A+ serum (Valley BioMedical, US) under standard conditions^77^. The ItG-ICAM-1 parasite line^78^, IT4var19 parasite line^50^ and HB3var03 parasite line^31^ were synchronized by alternating sorbitol treatment and gelatin selection. Mature trophozoite-stage IE (16–36 hrs post-invasion) or schizont-stage IE (>40 hrs and segmented) were enriched via magnet-column purification before being incubated with endothelial cell monolayers for 4–6 hrs or 12–20 hrs (overnight) at ratios ranging from 8–12 (10^6^) to 80–120 (10^7^) IE per endothelial cell. All parasite lines were regularly screened for mycoplasma negativity by PCR-based VenorGeM detection kit (Sigma, MP0025).

### Permeability assays

Endothelial permeability assays were measured using xCELLigence system from ACEA Biosciences. THBMEC and primary HBMEC were grown in EGM-2 (5% FBS) for 3–4 days to reach confluence in 96 well plates on integrated electronic sensors. The xCELLigence instrument measures real time changes in transendothelial resistance (TER) by electric cell-substrate impedance sensing (ECIS). For thrombin-induced barrier permeability assays, the media covering the cell monolayers was first changed to the experimental media condition (EGM-2 with 5% FBS or serum-free EGM-2 media complemented with all growth supplements) for 2 hrs, followed by the addition of the experimental concentration of IE or nE O+ suspension for 2 hrs. For thrombin challenge, 5 nM thrombin (T6884, Sigma) was added 2 hrs after IE addition to HBMEC. Control wells received nE O+ alone and/or thrombin alone. To evaluate the barrier restoration kinetics, the ECIS measurements were recorded for 2–3 hrs following thrombin treatment. We expressed the activity of thrombin-induced permeability to the maximum disruption observed as the minimum Cell Index (CI) and calculated the Area Under the Curve (AUC) for each given recorded period. The best-fit slope (m) and goodness of fit (R^2^) values were calculated from the recovery phase starting (dash line) corresponding to the maximum disruption time point observed.

### Statistical analyses

Statistical analysis was performed using Prism (version 7, GraphPad Software Inc.). Apoptosis data were compared using one-way ANOVA, Kruskal-Wallis non-parametric t test corrected by method of Benjamini, Krieger and Yekutieli. ELISA data were analyzed by the Mann-Whitney non-parametric unpaired, one-tailed t test. Permeability assays were compared using one-way ANOVA, Kruskal-Wallis non-parametric t test followed by Dunn’s Test for multiple comparisons. For each condition, nE was used as control.

## Supplementary information

Supplemental material

## Data Availability

All data generated or analyzed during this study are included in the published article (and its Supplementary Information Files).
