# Supplementary material for: Interplay of Plasmodium falciparum and thrombin in brain endothelial barrier disruption
Source: Sci Rep. 2019 Sep 11;9:13142. doi: 10.1038/s41598-019-49530-1 (PMC6739390; doi:10.1038/s41598-019-49530-1)
Supplement: Supplementary file 1 — Supplemental material [file 41598_2019_49530_MOESM1_ESM.pdf]

Interplay of *Plasmodium falciparum* and thrombin in brain endothelial disruption

Marion Avril[1], Max Benjamin[1], Mary-Margaret Dols[1], Joseph D. Smith[1,2*]

Supplemental Methods

Analysis of the primary brain endothelial cell barrier permeability by xCELLigence.

To study the permeability properties of the primary HBMEC using the xCELLigence device, cells were seeded 48 to 96 hrs prior the experiment to ensure the formation of a confluent monolayer (cell index CI measured every 2 hrs). If necessary, the cells were pre-stimulated by adding 10 ng/ml of TNFα 24 hrs prior to the addition of parasites and thrombin. Two hrs prior to parasite addition, the 200 μl content of the well were exchanged by either 100 μl complete media EGM-2 containing 5% FBS (EGM-2 +5%FBS) or no serum media (EGM-2 -FBS) (CI measured every 5 min). Near the end of this 2 hrs period, the baseline (control cells with EGM-2 -FBS media) was set and the CI was normalized to this time point. Then, we added either 50 μl of nE or IE concentrated to 10^6 (low) or 10^7 (high) total, which corresponds respectively to 8-12 or 80-120 IE/HBMEC (CI measured every 2 to 5min). After 2 or 3 hrs co-culture with HBMEC, 50 μl of either EGM-2 media alone or 5 nM thrombin was added (CI measured every minute). After a 2 hrs incubation, 200 μl of media was replaced by a pre-warmed *Pf* culture media containing 10% human sera and 2% hematocrit of normal erythrocytes (nE) (CI was measured every 10 to 30 min for the following 24 to 72 hrs).

To study the effect of parasites and thrombin on cell index, we recorded the maximum effect of barrier disruption defined as the minimum normalized cell index (CI) obtained within the 2 hrs period

following the addition of nE or IE or thrombin. We also calculated the area under the curve (AUC) within those 2 hrs to evaluate the recovery period.

Supplementary Figures

Figure S1. Immortalized THBMEC versus primary HBMEC endothelial cells. (A) Quantification of receptor expression by flow cytometry. Specific antibody (dark grey histograms) and isotype control antibody (light grey histograms). The median fluorescence intensity (MFI) is indicated in each panel. Two brain endothelial cell types HBMEC (ACBRI376, primary brain endothelial cells) and THBMEC (immortalized brain endothelial cells) were analyzed. A representative histogram from N = 3 independent experiments. (B) An xCELLigence assay was performed on THBMEC (grey traces) and primary HBMEC (black traces). The insets show the initial growth period with error bars for triplicate wells (left side) and the cells response to thrombin challenge after baseline normalization (right side). (C) A thrombin dose-titration experiment was performed in the presence of EGM-2 with 5% FBS (dotted lines) or EGM-2 with no serum (filled lines). The time of thrombin action to reach the minimum CI is indicated next to each condition.

Figure S2. Flow cytometry analysis of cell surface receptor on live HBMEC. (A) Flow cytometry gating strategy: HBMEC were selected based on forward and side scatter, then identified from singlet live cells by using the Live/Dead fixable violet stain (Pacific Blue). Percent of positive gated cells was reported in Figure 1. (B) Representative histograms of specific antibodies (dark colored histograms) and isotype control antibody alone (light colored histograms) detecting each receptor expression on resting (orange histograms) or stimulated (blue histograms) live primary endothelial cells HBMEC.

Figure S3. Overview of xCELLigence assay. A representative recording from an xCELLigence assay showing the raw data and how the baseline and normalization were set for all experiments. (A) Raw data shows the traces over the 96 hrs of experimental study. The arrowheads indicate when media was changed, or experimental components were added. The green timeframe indicates the 2 hrs window when HBMEC were switched from complete EGM-2 culture medium into EGM-2 with no serum or 5% serum. The blue timeframe indicates the 2 hrs window after IE addition and the yellow timeframe indicates the 2 hrs window after addition of thrombin. (B) The same CI curves as above following baseline correction using the resting HBMEC in presence of nE. (C) The same CI curves as above following the normalization time point after ~2 hrs of media change (black arrow head at the end of the green timeframe).

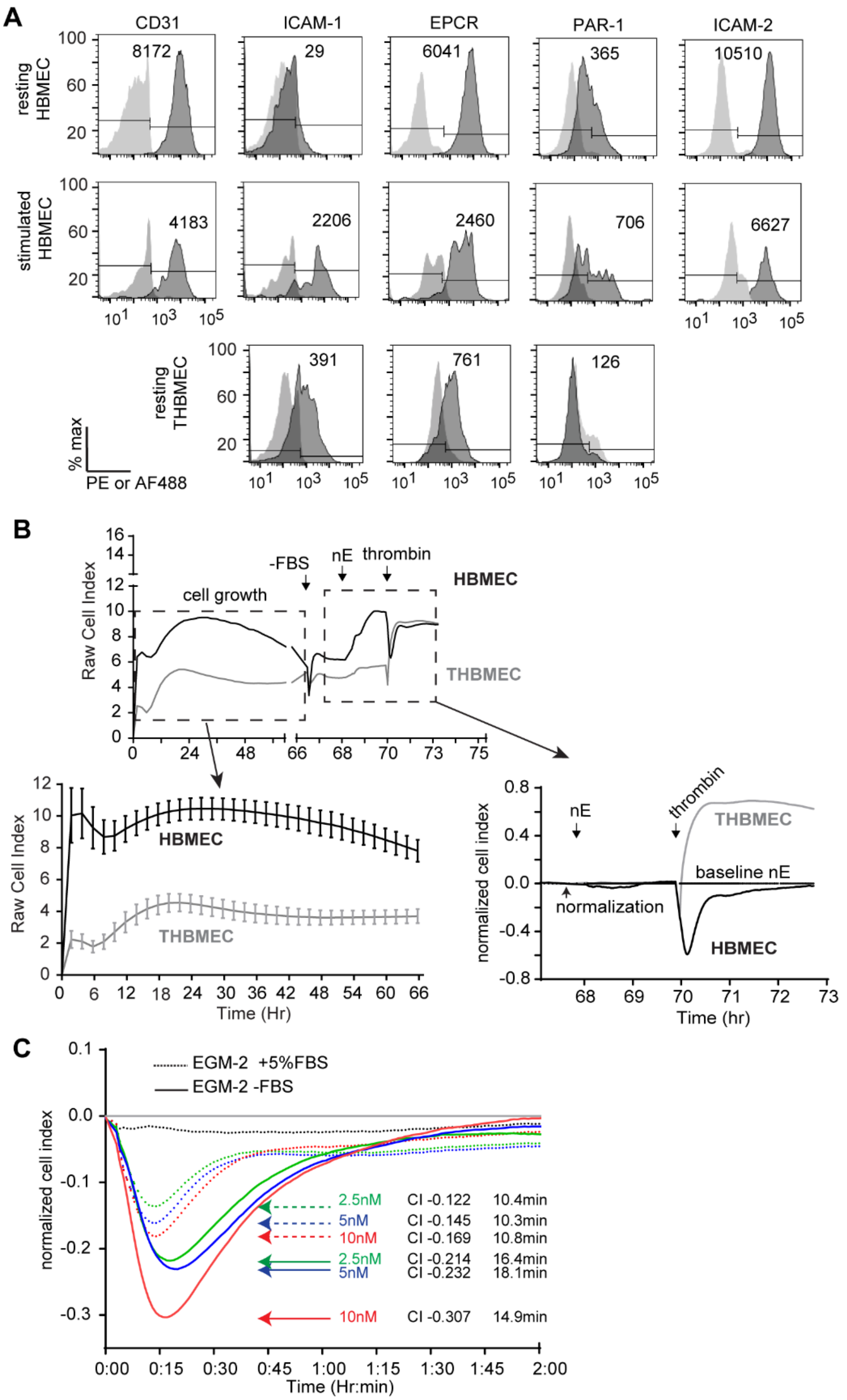

Figure S1. Immortalized THBMEC versus primary HBMEC endothelial cells. (A) Quantification of receptor expression by flow cytometry. Specific antibody (dark grey histograms) and isotype control antibody (light grey histograms). The median fluorescence intensity (MFI) is indicated in each panel. Two brain endothelial cell types HBMEC (ACBRI376, primary brain endothelial cells) and THBMEC (immortalized brain endothelial cells) were analyzed. A representative histogram from N = 3 independent experiments. (B) An xCELLigence assay was performed on THBMEC (grey traces) and primary HBMEC (black traces). The insets show the initial growth period with error bars for triplicate wells (left side) and the cells response to thrombin challenge after baseline normalization (right side). (C) A thrombin dose-titration experiment was performed in the presence of EGM-2 with 5% FBS (dotted lines) or EGM-2 with no serum (filled lines). The time of thrombin action to reach the minimum CI is indicated next to each condition.

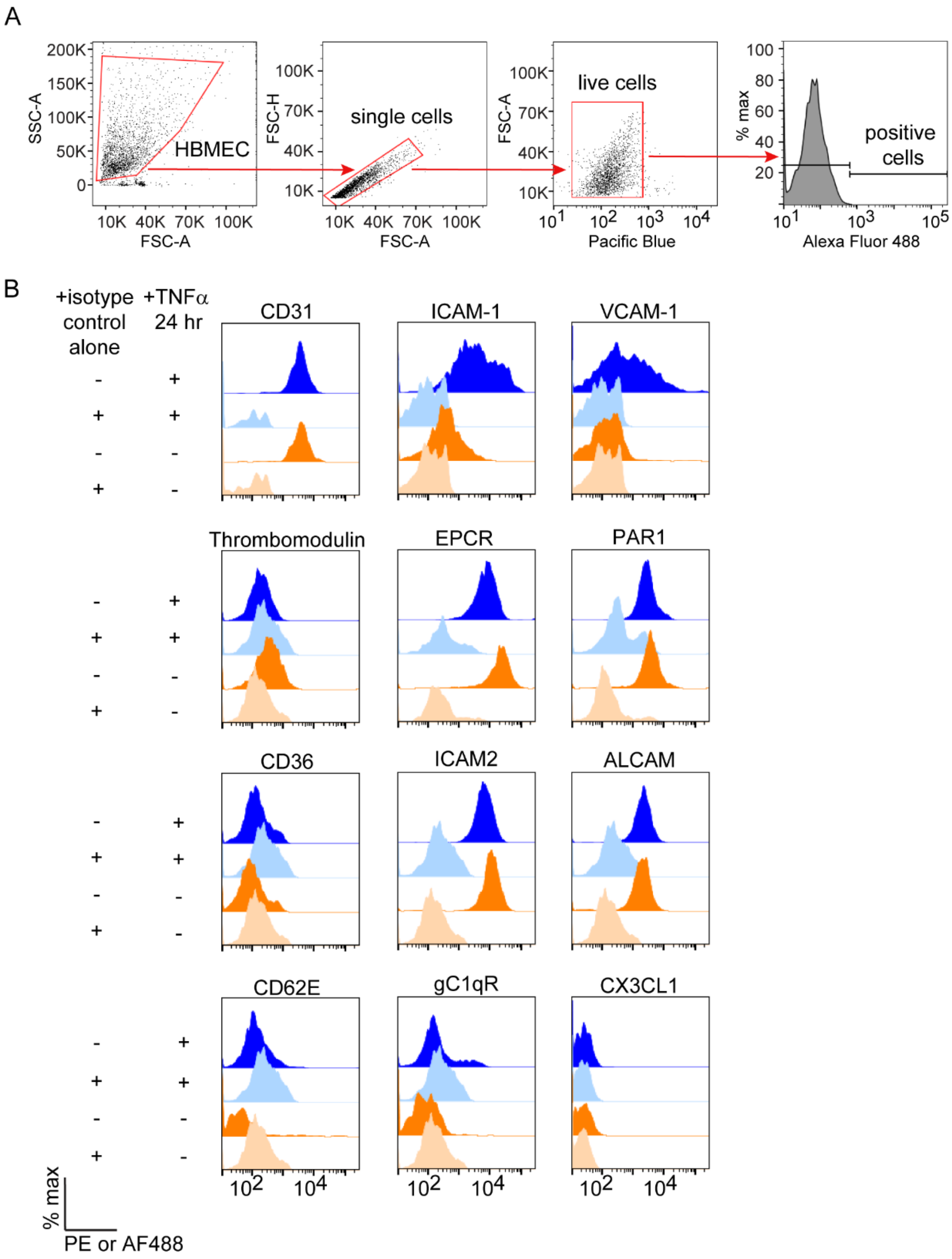

Figure S2. Flow cytometry analysis of cell surface receptor on live HBMEC. (A) Flow cytometry gating strategy: HBMEC were selected based on forward and side scatter, then identified from singlet live cells by using the Live/Dead fixable violet stain (Pacific Blue). Percent of positive gated cells was reported in Figure 1. (B) Representative histograms of specific antibodies (dark colored histograms) and isotype control antibody alone (light colored histograms) detecting each receptor expression on resting (orange histograms) or stimulated (blue histograms) live primary endothelial cells HBMEC.

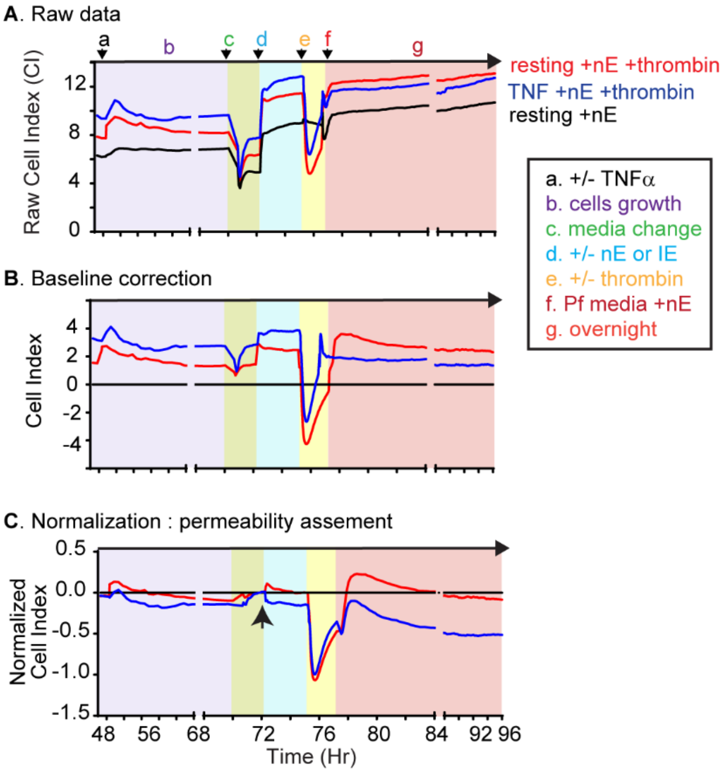

Figure S3. Overview of xCELLigence assay. A representative recording from an xCELLigence assay showing the raw data and how the baseline and normalization were set for all experiemnts. (A) Raw data shows the traces over the 96 hrs of experimental study. The arrowheads indicate when media was changed, or experimental components were added. The green timeframe indicates the 2 hrs window when HBMEC were switched from complete EGM-2 culture medium into EGM-2 with no serum or 5% serum. The blue timeframe indicates the 2 hrs window after IE addition and the yellow timeframe indicates the 2 hrs window after addition of thrombin. (B) The same CI curves as above following baseline correction using the resting HBMEC in presence of nE. (C) The same CI curves as above following the normalization time point after ~2 hrs of media change (black arrow head at the end of the green timeframe).
